# Concurrent ipsilateral fractures of acetabular posterior wall and femoral shaft associated with posterior hip dislocation: A case report and literature review of a rare injury

**DOI:** 10.1016/j.ijscr.2023.109035

**Published:** 2023-11-14

**Authors:** Reza Zarei, Mehdi Tavassoli, Mojtaba Baroutkoub, Sina Afzal

**Affiliations:** Department of Orthopedic Surgery, School of Medicine, Shahid Beheshti University of Medical Sciences, Tehran, Iran

**Keywords:** Hip injuries, Acetabulum fracture, Femur fracture, Hip dislocation, Case report

## Abstract

**Introduction:**

A floating hip injury involving the acetabulum and femur, often complicated by hip dislocation, necessitates a timely and appropriate management strategy to preserve the extremity and patient well-being.

**Case presentation:**

We present a case of a 20-year-old male with concurrent fractures of the acetabular posterior wall, a comminuted femoral shaft, and posterior hip dislocation. Reduction of the dislocated hip posed a challenge due to the femoral shaft fracture. We successfully employed an innovative technique, using pins proximal and distal to the shaft fracture in conjunction with a temporary external fixator, later replaced by an interlocking nail after hip reduction. Subsequently, we addressed the acetabular fracture through a posterior hip approach, enabling the patient to regain full weight-bearing capacity within a few months.

**Discussion:**

In managing concurrent injuries in a floating hip, particularly when a femoral shaft fracture is involved, innovative approaches, such as the one described in this study, are crucial for timely hip reduction. Following hip reduction, a series of surgeries are required to address the multiple lower extremity injuries, prioritizing those with the highest risk of adverse events and neurovascular complications.

**Conclusion:**

Urgent procedures for multiple fractures in orthopedic trauma surgery are pivotal for the best long-term outcomes. Prioritizing these urgent procedures, even through unconventional transient methods when conventional means are unavailable, can prevent long-term complications such as avascular necrosis. Effective and timely management is paramount for optimal patient recovery.

## Highlights

• Floating hip injuries, characterized by combined femoral fracture and acetabulum fracture, are infrequently encountered.
• Urgent hip dislocation reduction takes precedence over other procedures in the management of such injuries.
• The presence of a femoral fracture can introduce challenges when attempting to reduce the dislocated hip.

## 1 Introduction

Floating injuries of joints include a simultaneous injury of bones located on the two sides of a joint [1]. Floating hip injury, a concomitant fracture of the acetabulum and femur, is a rare type of lower extremities injury mainly seen in patients with high-energy polytrauma like motor vehicle accidents [2]. The term floating hip was coined first in the 1990s based on a series of cases with unstable pelvic or acetabular fractures associated with ipsilateral femoral fractures. It has been used frequently for similar cases since the injury makes the proximal lower extremity highly unstable, and surgical management needs appropriate prioritization of the injuries [2,3]. Although some experts in the field do not believe in using the term “floating” for hip in contrast to floating knee and floating elbow, which highly predispose the patient to serious neurovascular complications [4], publications on this injury still use the term to define the cases.

Two main classifications are available for floating hip; one, proposed by Liebergall et al. in groups A, defined as concomitant femoral fracture and ipsilateral open book pelvic fracture or unstable vertical shear, and B, defined as femoral fracture with acetabular fracture, which the latter is more known as a floating hip [2]. This classification was later developed to propose two sub-classes of the B type based on the mechanism of trauma, first as a posterior type injury including posterior acetabular fracture with diaphyseal femoral fracture at the same side, and second as a central type including central acetabular fracture and a proximal femoral fracture [1]. In the other classification, Muller et al. proposed three classes: (A) concomitant acetabular and femoral fractures, (B) concomitant pelvic ring and femoral fractures, and (C) combination of fractures of all three elements [4].

Although a variety of combinations of injuries in the floating hip have been reported in the literature, the presence of hip dislocation, besides the mentioned fractures, is a rare phenomenon that is reported in only a few studies [[5], [6], [7]]. The importance of such a combination of injuries is the challenge of reducing hip, which is vital to save the neurovascular compartments of the lower extremity and prevent adverse outcomes like avascular necrosis (AVN) of the femoral head [8] and seems to be difficult and even impossible in some cases [7]. In this study, we report a case with concurrent ipsilateral fractures of the acetabular posterior wall and femoral shaft associated with posterior hip dislocation that was successfully managed through a series of surgeries, with a further review and discussion of similar reported cases with such complicated injuries. This study has been reported in line with the SCARE criteria [9].

## 2 Case presentation

### 2.1 History and physical examination

We present a 20-year-old Persian male patient admitted with a left lower extremity deformity following a motor vehicle accident. The patient had no remarkable past medical history and used no specific medication at the time of referral. The patient had no history of drug abuse or smoking. At the time of physical examination, patient was conscious and had extreme pain in the pelvis and left thigh. The limb was shorter than the contralateral side and was in external rotation. The vital signs were in normal range except for mild tachycardia. Noticeable deformity in the left hip and thigh was observed. There was no evidence of a wound in the affected limb. The neurovascular examination of the left lower extremity was normal, and no loss of arterial pulses or neurological deficits were recorded. In the examination of other body parts, no evidence of severe trauma and injury was found, and the Injury Severity Score (ISS) was 21.

### 2.2 Work-up

**Fig. 1:**
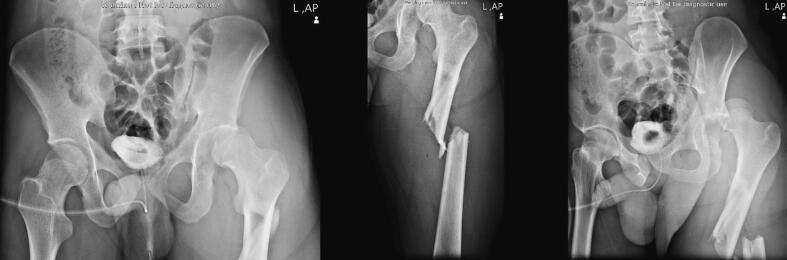
Plain anteroposterior radiography of the pelvis and femur shows a comminuted fracture of the femoral subtrochanteric region and a posteriorly dislocated hip.

**Fig. 2:**
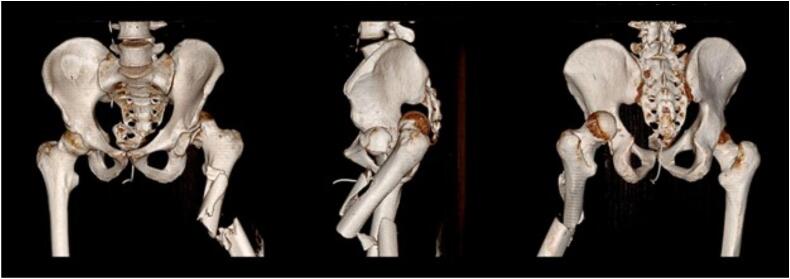
Pre-operative assessment of injuries by computed tomography imaging using 3D reconstruction.

After ruling out the possibility of any other trauma except for the left lower extremity, the patient underwent radiologic assessments by plain radiographic modalities to find out about the extent and type of injuries in the deformed extremity. In the pelvic X-ray (PXR), left hip posterior dislocation associated with a fracture in the posterior wall of acetabulum and comminuted subtrochanteric fracture were noted (Fig. 1). To make sure there is no non-displaced femoral neck fracture, 3-dimensional computed tomography (3D CT) scan of pelvis was done. The 3D CT scan revealed a posterior hip dislocation and a marginal fracture of the posterior acetabular rim, yet no other pathological findings (Fig. 2).

### 2.3 Surgical management

**Fig. 3:**
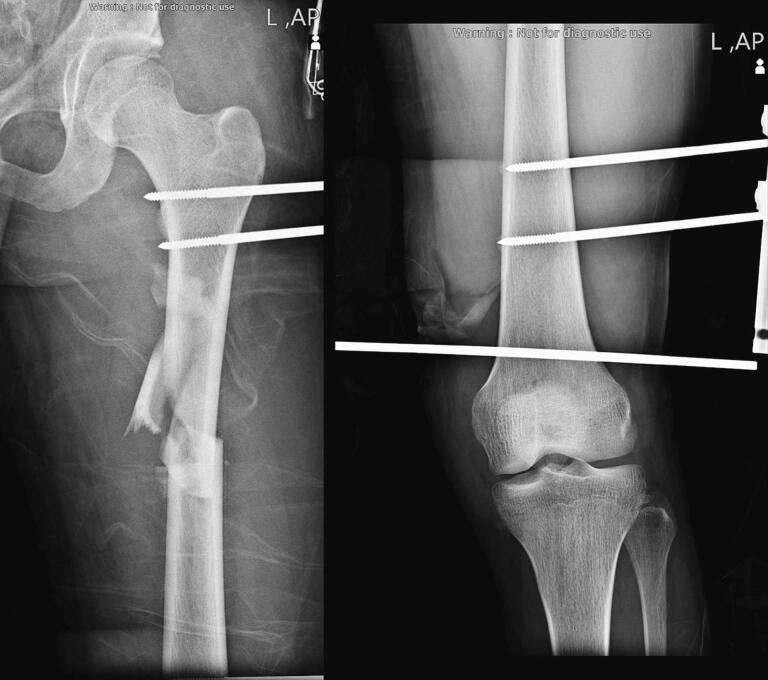
Inserted proximal pins to reduce hip dislocation using longitudinal traction and distal pins to align the femoral fracture using an external fixator temporarily.

The reduction of hip dislocation is the first-level priority in such cases to prevent AVN and other neurovascular complications like injuries to the sciatic nerve; however, due to the acetabular and femoral shaft fracture, the hip was in a floating state, and reduction was not possible by the routine Allis Maneuver. In the first surgery, in the supine position, in order to reduce the dislocated hip, two Schanz pins were inserted laterally into the subtrochanteric area to apply longitudinal traction. After hip dislocation reduction, two additional Shanz pins were inserted from the lateral aspect into the femoral shaft distal to the fracture. Following attaining relative alignment, the femur fracture was temporarily stabilized using an external fixator, which was made by connecting all four Schanz pins with two rods. Following that, based on the post-reduction hip instability discovered during the examination, a distal femur pin was inserted, and traction was administered. By the interventions in the first surgery, we successfully reduced the hip in 4 h after the initial trauma (Fig. 3). The post-operative evaluation of the neurovascular status of the extremity showed no deficits.

**Fig. 4:**
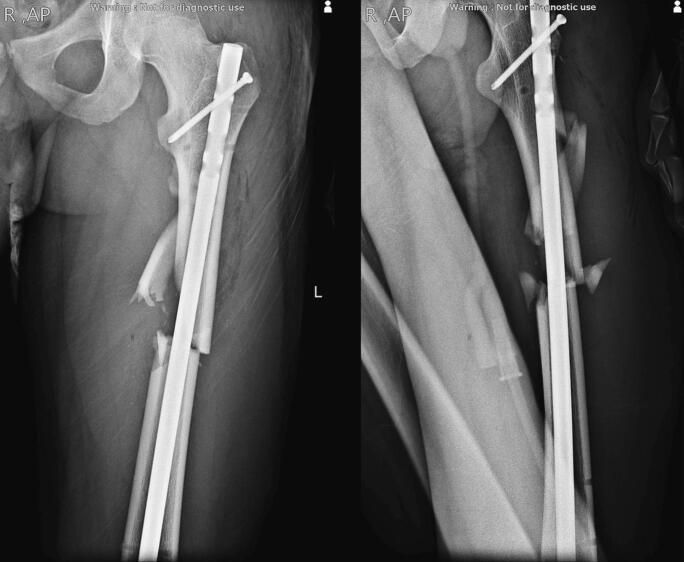
Definite fixation of the femoral shaft fracture with closed reduction and internal fixation using an antegrade intramedullary nail.

The second surgery was performed two days after the first stage, after the patient had undergone the necessary steps to be cleared from the general surgery service. During the second surgery, in the lateral position, the external fixator was replaced with an antegrade intramedullary nail (IMN) as the definite treatment of the femoral shaft fracture (Fig. 4).

After completing this stage, hip congruency was assessed using fluoroscopy, specifically in flexion, adduction, and internal rotation. The assessment revealed joint incongruency, signifying the necessity for corrective measures involving the joint capsule and the affected muscles located posterior to the hip. However, owing to the time-consuming nature of this procedure, it was deferred to a later stage. During this session, the distal femur pin was extracted, and traction was applied via skin traction.

During the third operation, due to the instability of the hip, the acetabular instability was addressed utilizing the Kocher-Langenbeck approach while ensuring the preservation of the sciatic nerve. This stage was performed in the prone position. The piriformis, superior gemellus, and obturator internus muscles were torn, and the piriformis tendon was incarcerated in the hip joint. After releasing the piriformis tendon and irrigating the hip joint to ensure the absence of any intra-articular fragment, the joint capsule and the short external rotator muscles were repaired. Subsequently, a hip stability examination revealed that the hip was stable.

### 2.4 Follow-up and outcomes

The patient received instruction on ambulation using a touch-bearing gait pattern with the assistance of two crutches and maintained this method of walking for a duration of three months. Subsequently, the patient progressively increased weight bearing until achieving full weight bearing. The patient was provided with a set of preventive measures, including the avoidance of extreme flexion and internal rotation, in order to prevent re-dislocation. These precautions were to be followed for one month. Four months after the operation, the patient was able to walk without crutches and had no pain at the femur fracture site. In the most recent follow-up examination of the patient, the range of hip movements was as follows: flexion: 90°, extension: 10°, internal rotation: 25°, external rotation: 40°, abduction: 35°, and adduction: 10°.

## 3 Discussion

In this study, we presented a complex injury of hip dislocation, acetabular fracture, and femoral shaft fracture that needed a multi-stage surgical management to reduce the dislocated hip and manage the acetabular and femoral fractures. Only a few similar cases have been reported in the literature, and other semi-similar cases are also available, which we will go through in the following sections to review the evidence on the presentation and management of such complicated injuries.

In the first most similar case, Rajasekaran and colleagues reported a 52-year-old male farmer who had a high-energy road traffic accident 7 h before admission and was resuscitated according to the advanced trauma life support protocols and was stable at the time of referral [5]. In radiologic examinations, the patient had a posterior acetabular wall fracture, ipsilateral femoral neck fracture and hip dislocation, and a middle third femoral shaft fracture on the left side. Also, the patient had a distal femur fracture on the opposite side. In this case, no hip reduction was done primarily, and the patient was taken for surgery 9 h after admission, during which the posterior wall of acetabulum was fixed by two contoured reconstruction plates. Next, the femoral head and shaft fractures were fixed using a reconstruction nail with two bolts. After three weeks, the patient experienced anterior dislocation of left hip due to myoclonic contractures, and later, the patient developed AVN of the femoral head, which led to hip replacement surgery eight months later, when the union on femoral shaft was completed. After that, the patient had no other complications, and healing of the injuries was pursued [5].

In the second most similar case, Tiedeken and colleagues reported a 35-year-old male patient involved in a high-speed biking accident, which was diagnosed with left hip posterior dislocation, posterior wall acetabular fracture, and transverse femoral diaphyseal fracture at the same side which was complicated by an engaging hill-sachs-type lesion of the femoral head [7]. Similar to the case we presented, the team managing this case used two Schanz pins proximal to the femoral shaft fracture with the aim of closed reduction of the dislocated hip by traction and related maneuvers. However, their efforts were ineffective due to the engaging femoral head lesion. This led to open reduction under direct visualization through a Kocher-Langenbeck approach and acetabulum fixation using two pelvic reconstruction plates. At the same surgery, the femoral shaft fracture was fixed with a retrograde reamed femoral nail. Following initial surgery, the patient underwent a fasciotomy procedure in response to the manifestation of compartment syndrome in the thigh region. Finally, the patient was discharged on postoperative day 22 and regained full weight-bearing 11 months later [7].

In the third most similar case, Duygulu and colleagues reported a 52-year-old male patient involved in a motor vehicle accident presented with his left leg four centimeters shorter and laterally rotated, on the radiographs, was diagnosed with left side hip dislocation, transverse-posterior wall acetabular fracture, femoral neck, and shaft fractures [6]. The patient also suffered from symphysis pubis diastasis and right distal radial fracture. The patient was transferred for surgery via a postero-lateral approach; first, acetabular fracture was reduced and fixed with a reconstruction plate, followed by antegrade intramedullary nailing to fix both femoral neck and shaft fracture. Also, the associated injuries of the pubis and radius were fixed with external fixators. Post-operatively, the patient started full-weight bearing after 12 weeks, and X-rays eight months after surgery showed union of all acetabular and femoral neck and shaft fractures [6].

Other similar reported cases with such concomitant injuries had more variations, and their management was accordingly different. These cases included a 45-year-old male patient with ipsilateral traumatic posterior hip dislocation, transverse-posterior wall acetabular fracture, and intertrochanteric fracture [10], a 20-year-old male patient with posterior hip dislocation and femoral head and shaft fractures at the same side [11], a 50-year-old male patient with traumatic posterior hip dislocation and femoral neck and shaft fractures [12], a traumatic anterior dislocation of hip and femoral shaft fracture at the same side in a 31-month-old female patient [13], ipsilateral posterior hip dislocation and femoral shaft fracture in a 24-year-old male patient [14].

A major component of patient management in the case presented in the current study was the temporary technique used to reduce the dislocated hip in spite of the femoral shaft fracture. Inserting pins proximal to the femoral shaft fracture in case of a concomitant hip dislocation was previously conducted and reported by Alhammoud and colleagues in 2016 on a similar case with posterior hip dislocation and ipsilateral femoral head and shaft fractures, which the dislocation was treated by closed reduction using a temporary external fixator on femur, followed by intramedullary nailing of the femoral shaft [15]. This technique was also used by Iftekhar and colleagues in 2020 on a patient with posterior hip dislocation and femur shaft fracture via a temporary external fixator on the femoral shaft to reduce the dislocated hip, followed by fixing the shaft fracture by IMN [14]. Overall, the successful management of these cases proposes this method as a viable option to reduce the dislocated hip in complex fractures of hip and femur before the end of its golden time to prevent adverse events like AVN and neurovascular complications.

## 4 Conclusion

Reducing the dislocated hip in floating hip injuries is a priority that might be challenging if associated injuries of the lower extremity, like femoral shaft fracture, happen at the same time. Benefitting from innovative techniques like what was proposed in this study, surgeons could successfully reduce the dislocated hip promptly to prevent adverse outcomes like AVN of the femoral head and other severe complications.

## Consent for publication

Written informed consent was obtained from the patient for publication and any accompanying images. A copy of the written consent is available for review by the Editor-in-Chief of this journal on request.

## Ethical approval

The Ethics Committee of the Shahid Beheshti University of Medical Sciences, School of Medicine, Tehran, Iran, to which the corresponding author belongs, does not require ethics review for case reports of medical procedures performed within the normal scope of care, if written consent is obtained from the individual patient.

## Funding

No funding.

## Author contribution

**Mehdi Tavassoli** conceptualized the study. **Sina Afzal** drafted the initial manuscript. **Mojtaba Baroutkoub** collected the patient's data. **Reza Zarei** revised the initial manuscript. All the authors read and approved the final version of manuscript.

## Guarantor

Sina Afzal.

## Research registration number

N/A.

## Conflict of interest statement

The authors declare that they have no competing interests.

## Data Availability

The material presented in this study is available from the corresponding author on a reasonable request.
